# Effects of Extracorporeal Shockwave Therapy on Pain and Mobility in Client-Owned Dogs with Refractory Elbow and Stifle Osteoarthritis: A Randomized Double-Blinded Trial

**DOI:** 10.3390/ani16040541

**Published:** 2026-02-09

**Authors:** Annika Klein, Elena V. Winkler, Yury Zablotski, Monika A. Mille, Frederik Volz, Susanne K. Lauer

**Affiliations:** 1 LMU Small Animal Clinic, Centre for Clinical Veterinary Medicine, Ludwig-Maximilians-Universitaet, 80539 Munich, Germany; annika.klein3597@gmail.com (A.K.); y.zablotski@med.vetmed.uni-muenchen.de (Y.Z.); monika.mille@lmu.de (M.A.M.); frederik.volz@chir.vetmed.uni-muenchen.de (F.V.); 2 Small Animal Clinic Oberhaching, 82041 Oberhaching, Germany; 3 Bogenhausen Veterinary Practice (Tierarztpraxis Bogenhausen Isabelle Heiss), 81929 Munich, Germany

**Keywords:** extracorporeal shockwave therapy, refractory osteoarthritis, stifle, elbow

## Abstract

Introduction: Extracorporeal shockwave therapy (ESWT) is used as an adjunctive treatment for canine osteoarthritis (OA), but its effects in dogs with treatment-refractory advanced disease remain unclear. This study compared the efficacy of one versus two sessions of focused ESWT administered approximately 28 days apart in dogs with refractory elbow or stifle OA. Methods: In this randomized, double-blinded clinical trial, twenty-four client-owned dogs with treatment-refractory elbow (*n* = 12) or stifle (*n* = 12) osteoarthritis received ESWT using an identical per-session protocol (X-Trode, 1000 pulses at 0.14 mJ/mm^2^; PulseVet-Zomedica, Ann Arbor, MI, USA), once (Group L) or twice (Group E). Orthopedic examination, goniometric and limb circumference measurements, and kinetic gait analysis (peak vertical pressure [PVP], vertical impulse [VI]) were performed on days 0, 28, and 56. Owner questionnaires (Canine Brief Pain Inventory [CBPI], Client Specific Outcome Measures [CSOM]) were collected on days 0, 28, 56, and 84. Data were analyzed using chi-squared tests, *t*-tests, and mixed effects models in R. Results: Age, weight, BCS, and radiographic osteoarthritis severity did not differ between groups at baseline. Improvement was small and limited to selected parameters. Vertical impulse and limb circumference increased more consistently in Group E, whereas peak vertical pressure increased in both groups, including before ESWT in Group L. No sustained or treatment-associated improvement was detected in symmetry variables or joint range of motion. Owner-reported outcomes showed variable patterns without consistent treatment effects. ESWT was well tolerated, and no major adverse events occurred. Conclusion: ESWT produced modest, inconsistent improvements in dogs with treatment-refractory OA, with slightly more consistent effects following two sessions. Therapeutic efficacy appeared limited in this end-stage population.

## Simple Summary

This randomized, double-blinded clinical trial evaluated whether one or two sessions of extracorporeal shockwave therapy (ESWT) improve pain and mobility in dogs with advanced, treatment-refractory elbow or stifle osteoarthritis. Both owner-reported assessments and objective outcome measures were used to assess treatment effects. Overall, improvements were small and mainly limited to selected objective measures, with dogs receiving two ESWT sessions showing slightly more consistent changes. However, ESWT did not result in substantial or sustained functional improvement in this severely affected population. Further studies with larger cohorts, optimized treatment protocols, and longer follow-up are needed to better define the role of ESWT in the management of canine osteoarthritis.

## 1. Introduction

Osteoarthritis (OA) is a common, progressive, and debilitating joint disorder in dogs, characterized by chronic synovial inflammation, degeneration of articular cartilage, and pathological changes in periarticular and subchondral bone [1,2]. Affected dogs commonly exhibit lameness, restricted range of motion (ROM), activity-related pain, and reduced mobility, all of which contribute to a decline in quality of life [3,4,5]. Epidemiological data suggest that OA affects up to 20% of adult dogs and as many as 80% of geriatric dogs, with the stifle and elbow joints being among the most frequently affected sites [6,7,8]. Cranial cruciate ligament rupture and elbow dysplasia are common underlying conditions predisposing to secondary osteoarthritic degeneration [9,10,11,12,13]. Despite advances in surgical and pharmacologic treatment, controlling disease progression and achieving long-term pain relief remain clinical challenges [9,10,11,13,14].

In dogs treated with tibial plateau leveling osteotomy (TPLO), radiographic progression of OA has been reported in up to 76% of patients as early as one year after TPLO, despite surgical stabilization [10]. One long-term follow-up study reported that approximately one-third of these dogs experience persistent pain [11]. Similar patterns are observed in dogs with elbow dysplasia, where lameness during routine activity is frequently observed [13]. In contrast to total hip replacement, which is well established and generally associated with favorable outcome in dogs [15,16], joint replacement for the elbow and stifle remains limited due to lower availability of suitable implants and inconsistent long-term success [15,17,18]. Elbow or stifle arthrodesis may be considered when replacement is not feasible or has failed, but postoperative outcomes are frequently unsatisfactory, and complication rates are high, limiting its value to salvage situations [19,20]. As a result, management of advanced OA in the elbow and stifle typically relies on conservative multimodal strategies [21,22]. Core components include body weight optimization [23], controlled exercise [24,25] and pharmacologic pain control, primarily with non-steroidal anti-inflammatory drugs [26,27,28,29,30]. Adjunctive measures, such as nutraceuticals [31], environmental modifications, and active caregiver participation, may further support long-term management [21].

Alternative strategies become increasingly relevant when conservative management fails to provide adequate relief [21,22,24]. Physical therapy constitutes a central element of multimodal OA management and aims to preserve joint function and reduce chronic pain [21,22,24,32]. The discipline encompasses manual techniques, therapeutic exercises, and modalities, such as electrotherapy, therapeutic ultrasound, low-level laser therapy, and extracorporeal shockwave therapy (ESWT) [21,22,32,33,34,35,36,37,38,39,40]. Among these modalities, ESWT has gained substantial popularity because of short treatment duration, a favorable safety profile and the potential for sustained therapeutic effects [22,32,36,37,38,41,42,43].

Extracorporeal shockwave therapy delivers high-pressure acoustic waves to target musculoskeletal tissues, thereby activating cellular responses through mechanotransductive signaling pathways [44,45]. Experimental and clinical data in human and veterinary medicine indicate analgesic and chondroprotective effects mediated through modulation of inflammatory activity, promotion of angiogenesis, and selective degeneration of nociceptive nerve fibers in osteoarthritic joints [36,37,38,41,42,46,47,48,49,50,51,52]. Clinical studies in dogs with hip and elbow OA describe functional improvements following ESWT, although findings remain inconsistent [38,41,42]. Variation in disease severity, underlying etiology, and treatment parameters, including energy flux density, pulse number, session intervals, and the use of focused versus radial devices, likely contributes to the heterogeneous outcomes [36,37,38,41,42]. For instance, two focused ESWT sessions after TPLO yielded improved lameness scores in dogs with chronic stifle OA [37], whereas a protocol of three sessions at three-week intervals did not demonstrate significant benefit [36]. Lack of protocol standardization limits meaningful comparison across studies and complicates clinical translation [49,53]. Evidence supporting the efficacy of ESWT in dogs with treatment-refractory OA remains limited. Available studies have largely focused on earlier disease stages or pharmacologic interventions [29,38].

The present randomized, double-blinded clinical trial evaluated the efficacy of focused electrohydraulic ESWT delivered with a commercially available device in client-owned dogs with advanced treatment-refractory OA of the elbow or stifle joint. The study compared the outcomes of one versus two treatment sessions using objective gait analysis and owner-reported assessments of pain and mobility. We hypothesized that two ESWT sessions administered 28 days apart would produce significantly greater improvements in functional outcome and pain reduction than a single session.

## 2. Materials and Methods

### 2.1. Study Design

This prospective, randomized, and double-blinded study was performed at LMU Small Animal Clinic from May 2022 to May 2024 (Centre for Clinical Veterinary Medicine, LMU Munich, 80539 Munich, Germany). The protocol for this study was approved by the Ethics Committee of the Centre for Veterinary Medicine, LMU Munich, Germany (approval number: 298-23-01-2022, date of approval: 30 March 2022), and all dogs were treated accordingly. Written informed consent was obtained from each dog owner upon entry into the study.

### 2.2. Inclusion Criteria and Study Schedule

Twenty-four dogs of any sex or breed with clinical signs of chronic pain and lameness due to OA of the elbow or stifle joint, which did not respond adequately to previous conservative treatments, were included in this study [54].

Dogs with a mean Canine Brief Pain Inventory (CBPI) score of ≥2 or Client Specific Outcome Measures (CSOM) score ≥ 1 were enrolled in this study [55,56,57]. Any potential surgical intervention for cranial cruciate ligament rupture, elbow dysplasia or an articular fracture must have been performed ≥ 1 year before study entry. Baseline data collection was performed at enrollment, including sex, age, body weight, body condition score (BCS), and the date of surgery. All dogs were examined by one of the authors (E.W., F.V., and S.L.) at day 0 (baseline) and at follow-up visits scheduled for approximately 28 and 56 days later, depending on owner availability. All clinical assessments were performed by the same veterinarian whenever possible. In three dogs, a single examination was conducted by an alternate examiner due to unforeseen circumstances. General and orthopedic examination, standard goniometric measurements of the affected joint, muscle circumference measurement of the affected extremity, as well as kinetic gait analysis in walk and trot were performed in the same order for all clinic visits.

### 2.3. Exclusion Criteria

#### 2.3.1. Clinical Symptoms

Participants were excluded if they experienced pain in the spine, hip, shoulder, tarsal or carpal joints attributable to other musculoskeletal or neurologic disorders. Dogs with a history or clinical signs suggestive of a current meniscal injury, including meniscal clicks identified during orthopedic examination, were not eligible. Dogs with suspected septic arthritis, osteomyelitis, periarticular wound infection, or other joint disorders associated with secondary OA for which surgical intervention was indicated but not yet performed were not included.

#### 2.3.2. Medications

Dogs were excluded if they had received steroidal anti-inflammatory drugs within six weeks before enrollment, and a sustained anti-inflammatory effect was anticipated based on the specific glucocorticoid administered. Intra-articular injections into the affected stifle or elbow joint within the preceding 90 days also constituted an exclusion criterion. Non-steroidal anti-inflammatory drugs were permitted if the drug type, dosage, and administration schedule remained unchanged for several weeks prior to enrollment and throughout the study period. Monoclonal antibodies were permitted only if dogs had received at least two administrations before study inclusion, with consistent dosing intervals maintained during the study.

### 2.4. Randomization and Blinding

Enrolled dogs were randomly assigned in a 1:1 ratio to one of two treatment groups, each comprising six dogs affected by elbow OA and six by stifle OA. The early treatment group (Group E) received focused ESWT (ESWT) at baseline (day 0) and approximately 28 days later, whereas the late treatment group (Group L) underwent a sham treatment at baseline followed by ESWT approximately 28 days later. Both owners and clinical evaluators were blinded to group allocation for the duration of the study.

### 2.5. Radiographic Evaluation

Two-dimensional radiographic examinations of the affected and contralateral joint for suspected bilateral OA were conducted at baseline to confirm the presence of OA and to exclude other disorders. Orthogonal radiographs of the affected stifle joint were evaluated by the first author (A.K) using the Mager scoring system to assess OA severity across 15 anatomical landmarks [58]. For the elbow joint, mediolateral radiographs in a neutral 120° position and craniocaudal views were obtained. The severity of elbow OA was classified by the first author (A.K) using the International Elbow Working Group (IEWG) arthritis classification system [59].

### 2.6. Canine Brief Pain Inventory (CBPI) and Client Specific Outcome Measures (CSOM)

Patient owners received the CBPI and CSOM questionnaires [55,56,57,60] by email before the initial evaluation and at follow-up time points on days 14, 28, 56, and 84.

The mean CSOM total score (twelve items, mean score: 0–4), mean CBPI total score (CBPI Total: ten items, total score: 0–100, mean score: 0–10), mean CBPI pain score (CBPI Pain: four items, total score: 0–40, mean score: 0–10), and mean CBPI interference activity score (CBPI Activity: six items, total score: 0–60; mean score: 0–10) were calculated for all assessment days. All questionnaires were completed using Microsoft forms.

### 2.7. Standard Goniometric Evaluation

Goniometric measurements were conducted by the blinded examiners immediately following the orthopedic examination on days 0, 28, and 56. All evaluations were performed with the dogs in lateral recumbency and without sedation. For each joint, three repeated measurements were taken at maximal flexion and extension using a transparent plastic goniometer (Goniometer, KaWe-KIRCHNER& WILHELM GmbH + Co. KG., Asperg, Germany) aligned with established anatomical landmarks [61].

### 2.8. Limb Circumference Measurements

Muscle circumference of the treated limb was measured by the examiner using a Gulick II Tape Measure (Model 67020; Fitness Mart, Gays Mills, WI, USA), with the dog standing in a neutral, square, and relaxed position [62]. Measurements were taken at 70% of the length of the brachium or thigh, depending on whether the elbow or stifle joint was treated [63,64]. Consistent tape tension was maintained for all measurements, and care was taken to prevent distal slippage. At each assessment time point (days 0, 28, and 56), three consecutive measurements were obtained [62].

### 2.9. Kinetic Gait Analysis

Kinetic gait analysis was conducted using a pressure-sensitive treadmill (CanidGait^®^, Zebris Medical GmbH, Isny, Germany; sensitivity 0.5 N/cm^2^; pressure-sensitive surface: 163 × 41 cm) equipped with 9216 individually calibrated capacitive pressure sensors (spatial resolution: 0.4 cm) and a sampling frequency of 100 Hz [65]. Synchronized video recordings were obtained using three cameras (SYNCLightCam, Zebris Medical GmbH, Isny, Germany) positioned behind and on both sides of the treadmill, and subsequently analyzed using proprietary software (WinFDM Version 1.2.2). Following a brief acclimatization period, gait analysis was performed during both walking and trotting. Treadmill velocity was individually adjusted for each dog and held constant across all assessment time points. For both walk and trot, three valid trials, each comprising five consecutive steps per limb, were selected using video recordings to confirm adequate posture. A trial was considered valid if the dog maintained a steady pace with minimal head or body movement and no leash tension. Total evaluation time per session did not exceed 15 min.

Peak vertical pressure (PVP; Newtons) and vertical impulse (VI; N·s) were calculated using proprietary analysis software (Animal Analysis Suite, Version 2.8) and expressed as a percentage of body weight (%BW) [3,65]. For both walk and trot, the mean symmetry index of peak vertical pressure (SI_PVP_) between the treated and contralateral limbs was calculated as previously described (SI = [(PVP contralateral limb—PVP treated limb)/((PVP contralateral limb + PVP treated limb) × 0.5)] × 100) [66,67]. An SI_PVP_ of zero indicates a perfectly symmetric gait. Cut-off values for diagnosing hindlimb lameness were defined as greater than 9% at the walk and greater than 6% at the trot [68]. The mean ratio of peak vertical pressure between the forelimb (FL) and hind limb (HL) on the treated side (Ratio_PVP(FL:HL)_) was also calculated and compared with the expected forelimb-to-hindlimb pressure ratio of approximately 1.60 reported in healthy dogs [68,69,70,71].

### 2.10. Extracorporeal Focal Shockwave Therapy and Sham Treatments

Following completion of all clinical evaluations, ESWT was administered to the affected joint according to group allocation at baseline (day 0), a follow-up visit approximately 28 days later for the early treatment group (Group E), and the approximate day-28 follow-up visit for the late treatment group (Group L). In dogs with bilateral OA, the most severely affected joint was treated based on clinical orthopedic examination and objective gait analysis [36]. To maintain blinding, all treatments were performed in the absence of the owners and assessors (E.W., F.V., and S.L.) by the first author (A.K.), who was not involved in outcome assessments. A focused electrohydraulic shockwave device (PulseVet with Xtrode; Zomedica, Ann Arbor, MI, USA) was used for all ESWT applications. The treatment protocol was standardized across all sessions and implemented in accordance with the manufacturer’s recommendations and previous studies investigating the effects of ESWT in dogs with stifle and elbow OA [37,38].

Before treatment, hair was clipped over the lateral and medial aspects of the selected joint, and coupling gel (Aquasonic 100, PARKER, Fairfield, NJ, USA) was applied to ensure optimal acoustic transmission. Treatments were performed with the dog in lateral recumbency. The Xtrode was positioned at a 90° angle to the joint surface and moved in a slow circular motion. Each session consisted of 1000 shockwave pulses, 500 applied to the lateral aspect and 500 to the medial aspect of the joint. Energy flux density and pulse frequency were gradually increased over the first 50 to 100 pulses, up to a maximum of 0.14 mJ/mm^2^, with final settings adjusted according to individual tolerance.

Sham treatments followed identical preparation and positioning protocols, including clipping and gel application. However, the Xtrode was not placed in contact with the skin, and no shockwaves were delivered during the 1000-pulse cycle.

Two days following the potential ESWT or sham procedure, owners were contacted by telephone or email to assess the occurrence of adverse events. Owners were specifically asked about observable changes in their dogs, including increased lameness, reduced weight bearing, altered activity level, local swelling, redness, warmth, bruising, increased licking or guarding of the treated limb, and any behavioral changes suggestive of discomfort. Minor adverse events were defined as transient, owner-observed signs of discomfort or local tissue reactions that resolved spontaneously within two weeks after treatment. Major adverse events were defined as persistent or severe clinical signs, including marked lameness, progressive swelling, signs of infection, or any adverse event requiring additional veterinary evaluation or medical intervention, with or without irreversible tissue damage, in accordance with previously published criteria adapted from human ESWT safety studies [72].

### 2.11. Statistical Analysis

The variables, including age, body weight, BCS, OA grade, CSOM, CBPI_Total_, CBPI_Pain_, CBPI_Activity_, SI_PVP_, and Ratio_PVP(FL:HL)_, were analyzed as absolute values. Goniometric measurements, limb circumference, PVP (expressed as percentage of body weight), and VI (expressed as percentage of body weight) were normalized to each dog’s baseline (day 0) and expressed as relative values (percentages).

Age, BCS, and Mager scores were analyzed using Student’s *t*-test, as these variables met assumptions of normal distribution and equal variance between groups. The chi-squared test was used to compare OA-grades.

All remaining parameters, including CSOM, CBPI, limb circumference, goniometry, PVP, VI, SI_PVP_, and Ratio_PVP(FL:HL)_, were analyzed using mixed-effects models to account for repeated measures across multiple time points. The individual dog was treated as the random effect in the model, and the groups and time points were modeled as an interaction. Before analysis, model assumptions were evaluated as follows: (1) normality of data for tests and of the residuals for models was assessed using the Shapiro–Wilk normality test, (2) the homogeneity of variances between groups was assessed using Levene’s test, and (3) heteroscedasticity (constancy of error variance) was assessed using the Breusch–Pagan test.

When model assumptions were met, generalized linear mixed effects models were applied (R package—lmer). When assumptions were violated, robust linear mixed effects models were used (R package—robustlmm). Linear and robust linear models were compared using performance quality indicators, including conditional and marginal coefficients of determination R2, the intraclass-correlation coefficient (ICC), and the root mean square error (RMSE). The model with the best overall fit was selected. All contrasts (differences) between groups at each time point and between time points within each group were assessed after model-fitting using estimated marginal means (R package—emmeans), with Tukey *p*-value correction for multiple comparisons. A 95% confidence level was applied to report uncertainty, and *p*-values < 0.05 were considered statistically significant. All statistical analyses were conducted using R (version R 4.2.1 (23 June 2022)).

## 3. Results

### 3.1. Demographics

Thirty-one dogs met the inclusion criteria and presented for the initial clinic visit. Seven dogs were excluded from the study due to loss to follow-up (*n* = 3), modification of the medication protocol during the study period (*n* = 1), inability to walk on a treadmill (*n* = 1), owner-initiated withdrawal (*n* = 1), or intolerance to shockwave therapy without sedation (*n* = 1).

The remaining study population consisted of 24 dogs, comprising four intact females, seven spayed females, four intact males, and nine neutered males. Breeds represented included Labrador Retriever (*n* = 8), mixed breed (*n* = 7), Rhodesian Ridgeback (*n* = 2), and one each of Golden Retriever, Australian Shepherd, Beauceron, Collie, German Shepherd, German Shorthaired Pointer, and Old English Bulldog.

Twenty dogs had undergone orthopedic surgery before study enrollment. The interval between surgery and study enrollment ranged from 357 to 2128 days. The mean interval between the first and second examination days was 28.25 ± 2.71 days in Group E and 30.75 ± 4.28 days in Group L. Between the second and third examination days, the mean interval was 27.33 ± 4.46 days for Group E and 30.75 ± 3.8 days for Group L. Underlying conditions included cranial cruciate ligament rupture (*n* = 11), elbow dysplasia (*n* = 7), and a distal femoral epiphyseal fracture (*n* = 1). Surgical procedures included tibial plateau leveling osteotomy (TPLO) of a single stifle joint (*n* = 7), including meniscectomy in two dogs and bilateral TPLO (*n* = 3), including meniscectomy in one dog. Additional surgical procedures were extracapsular suture stabilization (*n* = 1), femoral fracture repair (*n* = 1), arthroscopy with subtotal coronoidectomy of one elbow (*n* = 3) or both elbows (*n* = 3), ulnar ostectomy (*n* = 1), and removal of an ununited anconeal process (*n* = 1). Two dogs had received gold bead implantation in both elbows (*n* = 2), and one dog had previously undergone radiation therapy to an elbow joint.

Before study inclusion, all dogs received medical management for osteoarthritis. Bedinvetmab was discontinued weeks to months earlier in three dogs due to insufficient clinical response. Non-steroidal anti-inflammatory drugs were administered on an as-needed basis in three dogs, with owners reporting only mild symptomatic relief and concerns regarding adverse effects. Consequently, these medications were reserved for acute exacerbations. All dogs had received NSAIDs at some point before enrollment, although in several cases treatment was discontinued because of limited perceived efficacy or adverse effects, and detailed information on treatment duration and reasons for discontinuation was not consistently available. Two dogs had previously received additional analgesics (pregabalin in one dog and tramadol in another) in combination with NSAIDs. However, these medications were discontinued several weeks before study enrollment due to insufficient clinical effect and were not administered during the study period.

At the time of enrollment, seven dogs were receiving analgesic medications, including bedinvetmab (*n* = 5), meloxicam (*n* = 1) and carprofen (*n* = 1). Based on the owner’s report, dosages and administration schedules of these medications remained unchanged throughout the study period. In addition, twenty dogs were receiving dietary supplements, such as glucosamine, chondroitin, omega-3 fatty acids, evening primrose oil, or devil’s claw. Three dogs received additional physiotherapy, including underwater treadmill training, massage therapy, and low-level laser therapy, at intervals of two to four weeks. The treatment protocol and timing remained consistent throughout the study period.

Upon study entry, mean age (Group E: 8.05 ± 2.47 years, Group L: 7.56 ± 2.82 years), body weight (Group E: 31.53 ± 7.04 kg, Group L: 31.99 ± 7.13 kg), and body condition score (Group E: 5.42 ± 0.95, Group L: 5.58 ± 1.11) did not differ between groups. Body weight reduced significantly by 0.37 ± 0.12 kg in Group L from day 0 to day 56 (*p* = 0.006), whereas in Group E did not change significantly across the study period.

In Group E, mild, moderate, and severe stifle OA were present in two, three and one dogs, respectively, as graded using the Mager scoring system for orthogonal radiographs [58]. Elbow OA in this group was classified as moderate in two dogs and severe in four dogs according to the International Elbow Working Group arthritis grading system for two-dimensional radiographs [59]. In Group L, five dogs had mild stifle OA, and one had moderate stifle OA. Elbow OA in this group was classified as mild in one dog and severe in five dogs using the same radiographic criteria. Bilateral joint involvement was present in three dogs in Group E and six dogs in Group L.

Mean elbow OA severity scores (Group E: 2.67 ± 0.47, Group L: 2.67 ± 0.75) and Mager scores (Group E: 40.67 ± 6.67, Group L: 34.67 ± 4.53) did not differ significantly between the two groups.

Extracorporeal shockwave therapy was well tolerated, and predominantly mild adverse events were observed. A mild, transient deterioration of lameness was reported in one elbow patient in Group E after the first treatment and resolved within three to five days. In one elbow patient in Group L, mild, transient erythema and a deterioration of lameness were reported after the first treatment. While erythema resolved within two days, worsening of lameness persisted for approximately 14 days following treatment. In this dog, objective gait analysis during walk demonstrated a deterioration of approximately 10% at Day 28, when only sham treatment had been applied at baseline, and a further deterioration of approximately 20% at Day 56. Resolution within the predefined short-term adverse event window was therefore not observed in this case. A causal relationship with ESWT cannot be established. In addition, two stifle patients in Group E exhibited mild discomfort and a transient increase in lameness, occurring after each treatment session in one dog and after the first treatment session in another, both resolving within two days. No adverse event required medical intervention, and no major adverse events occurred.

### 3.2. CBPI and CSOM

The results of the CBPI and CSOM across all assessment points are summarized in Table 1. Owners completed the CBPI and CSOM questionnaires within one to five days of distribution. Baseline questionnaires were completed by the day of study enrollment (Day 0) and were therefore considered representative of baseline status for analysis. Mean CSOM did not differ significantly between Group E and Group L at any time point. In Group E, the mean CSOM did not differ significantly between any time points. In Group L, mean CSOM decreased significantly from day 0 to day 28 (*p* = 0.044), but no other significant changes were observed between time points.

Mean CBPI_Total_ did not differ significantly between Group E and Group L at any time point. In Group E, the mean CBPI_Total_ did not differ significantly between any time points. In Group L, the mean CBPI_Total_ decreased significantly from day 0 to day 28 (*p* = 0.010) and from day 0 to day 56 (*p* = 0.039), but no other significant changes were observed between time points.

The mean CBPI_Pain_ did not differ significantly between Group E and Group L at any time point. In Group E, the mean CBPI_Pain_ decreased significantly from day 0 to day 28 (*p* = 0.019) and from day 0 to day 56 (*p* = 0.001), but no other significant changes were observed between time points. In Group L, mean CBPI_Pain_ did not differ significantly between any time points.

The mean CBPI_Activity_ did not differ significantly between Group E and Group L at any time point. In Group E, the mean CBPI_Activity_ did not differ significantly between any time points. In Group L, the mean CBPI_Activity_ decreased significantly from day 0 to day 14 (*p* = 0.019), from day 0 to day 28 (*p* = 0.001), and from day 0 to day 56 (*p* = 0.002), but no other significant changes were observed between time points. This section may be divided by subheadings. It should provide a concise and precise description of the experimental results, their interpretation, and the experimental conclusions that can be drawn.

### 3.3. Standard Goniometric Evaluation and Limb Circumference Measurements

Goniometric and limb circumference data for both groups at all time points are presented in Table 2.

The mean joint ROM, extension, and flexion did not differ significantly between Group E and Group L at any time point, except for day 28, when joint extension was significantly higher in Group L than in Group E (*p* < 0.05).

In Group E, the mean ROM, extension, and flexion did not differ significantly between any time points. In Group L, mean ROM increased significantly from day 0 to day 28 (*p* = 0.017), and mean extension increased significantly from day 0 to day 28 (*p* = 0.0005) and day 56 (*p* = 0.010). The mean flexion did not differ significantly between any time points.

The mean limb circumference did not differ significantly between Group E and Group L at any time point. In Group E, the mean limb circumference increased significantly from day 0 to day 56 (*p* = 0.012) and from day 28 to day 56 (*p* = 0.036). In Group L, mean limb circumference did not differ significantly between any time points.

### 3.4. Kinetic Gait Analysis

Kinetic gait analysis values in walk and trot of both groups at each time point are illustrated in Table 3.

#### 3.4.1. Walk

The mean PVP and VI did not differ significantly between Group E and Group L at any time point. The mean PVP did not differ significantly between any time points in either group. In Group E, the mean VI did not differ significantly between any time points. In Group L, mean VI decreased significantly from day 28 to day 56 (*p* = 0.0004), and no other significant changes were observed.

The mean SI_PVP_ did not differ significantly between Group E and Group L at any time point. In Group E, the mean SI_PVP_ increased significantly from day 0 to day 28 (*p* = 0.023), with no other significant changes. In Group L, the mean SI_PVP_ decreased significantly from day 0 to day 28 (*p* = 0.010), with no other significant changes.

The mean Ratio_PVP(FL:HL)_ did not differ significantly between Group E and Group L at any time point, except at day 56, when Group E showed significantly higher values (*p* = 0.033). In Group E, the mean Ratio_PVP(FL:HL)_ did not differ significantly between any time points. In Group L, the mean Ratio_PVP(FL:HL)_ decreased significantly from day 0 to day 56 (*p* = 0.014) and from day 28 to day 56 (*p* = 0.042).

#### 3.4.2. Trot

The mean PVP and VI did not differ significantly between Group E and Group L at any time point. In Group E, the mean PVP increased significantly from day 0 to day 56 (*p* = 0.012). In Group L, the mean PVP increased significantly from day 0 to day 28 (*p* = 0.048) and from day 0 to day 56 (*p* = 0.0002). The mean VI increased significantly in Group E from day 0 to day 56 (*p* = 0.044), while no significant changes were observed in Group L.

The mean SI_PVP_ did not differ significantly between Group E and Group L at baseline. On day 28 (*p* = 0.037) and day 56 (*p* = 0.024), Group E had significantly higher SI_PVP_ values than Group L. In Group E, the mean SI_PVP_ increased significantly from day 0 to day 28 (*p* = 0.001) and from day 0 to day 56 (*p* = 0.0003). In Group L, mean SI_PVP_ did not differ significantly between any time points.

The mean Ratio_PVP(FL:HL)_ did not differ significantly between Group E and Group L at any time point. In Group E, the mean Ratio_PVP(FL:HL)_ decreased significantly from day 28 to day 56 (*p* = 0.0001). In Group L, the mean Ratio_PVP(FL:HL)_ increased significantly from day 0 to day 28 (*p* = 0.0003) and from day 0 to day 56 (*p* = 0.005).

## 4. Discussion

This randomized, double-blinded clinical trial evaluated the effects of one versus two sessions of focused electrohydraulic ESWT in dogs with advanced treatment-refractory elbow or stifle OA. Only small and inconsistent improvements were detected, and the overall magnitude of response remained limited across both groups. Differences between one and two treatment sessions were minimal, and the hypothesis that two sessions administered 28 days apart would lead to significantly greater improvements was supported only for selected kinetic parameters without evidence of consistent clinical relevance.

Dogs with treatment-refractory OA represent the most challenging patient subgroup, in which to achieve measurable improvement, and the limited treatment response observed in this study aligns with outcomes reported for other therapeutic interventions in similar populations. Multimodal analgesic therapy [29], orthobiologic treatments [73,74] and low-dose radiotherapy [75] have all yielded modest or inconsistent clinical benefits in dogs with moderate to severe OA.

The biological constraints characteristic of refractory disease, including long-standing pain, chronic inflammation, and marked osteophytosis, contribute to reduced therapeutic responsiveness. Salvage procedures for the elbow and stifle are limited by implant availability and inconsistent long-term outcomes [17,18,76], underscoring the scarcity of effective options for end-stage disease in dogs. Dogs with earlier-stage disease may allow clearer protocol comparisons, but ethical restrictions on withdrawing effective medications and the absence of an established ESWT washout interval constrain such designs. Consequently, extrapolation of the present findings to dogs with less advanced disease should be made cautiously and requires dedicated evaluation.

Changes in CBPI and CSOM were small and inconsistent across groups. Group-specific fluctuations, including changes before treatment in Group L, suggest that caregiver-related variation, expectation effects and natural day-to-day variability influenced these outcomes. The sensitivity, specificity, and minimal clinically important differences of clinical metrology instruments have not been specifically validated for patients with refractory OA, either in veterinary [55,77] or human medicine [78,79]. These factors limit the ability to determine whether the observed patterns reflect a true absence of treatment effect or insufficient responsiveness of the available instruments in severely affected dogs.

Joint range of motion did not improve following ESWT, and the only significant changes occurred before treatment, indicating these were unrelated to therapy. This finding aligns with previous reports suggesting that ESWT does not modify joint ROM in dogs with stifle [36] or elbow osteoarthritis [38]. In human medicine, patients with end-stage elbow or knee osteoarthritis exhibit limited capacity for improving joint excursion, even with structured rehabilitation, and gains are typically small, require prolonged therapy and rarely restore normal mobility [80,81,82,83,84]. These observations underscore the limited potential for meaningful improvement in passive joint motion in dogs with advanced structural restriction. Limb circumference increased significantly in the group receiving two ESWT sessions. Under controlled conditions in healthy dogs, Gulick tape measurements demonstrate low technical variability [63,64], although comparable reliability cannot be assumed in clinical populations with joint pain and muscle guarding. Based on previously published data, a technical intraobserver error of approximately 2% for thoracic and thigh girth measurements can be expected [62,85]. The observed increase of approximately 3%, therefore, slightly exceeded the anticipated technical measurement error and may reflect changes beyond measurement variability. Improvements in selected kinetic variables observed later in the study period are compatible with a delayed or time-dependent effect. However, given the study design and sample size, definitive conclusions regarding causality cannot be drawn, and it cannot be determined whether a similar increase would have occurred in the single-treatment group with longer follow-up.

Objective gait analysis yielded a more consistent basis for evaluating potential treatment effects, although improvements remained modest. Peak vertical pressure during trot increased in both groups, exceeding the 5% threshold proposed as clinically meaningful [86], but the presence of a significant increase before treatment in Group L indicates that not all changes can be attributed to ESWT. Dogs receiving two ESWT sessions demonstrated additional improvement in VI during trot, suggesting a cumulative or time-dependent effect with uncertain clinical importance. Symmetry indexes and forelimb-to-hindlimb pressure ratios did not show consistent patterns of improvement. This is consistent with findings in healthy dogs, in which inherent gait asymmetry and substantial trial-to-trial variability are present [87,88]. Interpretation was further complicated by bilateral joint disease in several dogs.

The limited treatment response observed in the present study is consistent with existing canine literature. A direct comparison between Group E and Group L at Day 28 revealed significant differences in joint extension, driven by improvement in Group L, and in the symmetry index during trot, reflecting deterioration of uncertain clinical relevance in Group E. Across studies, ESWT has produced modest short-term improvements in limb loading without restoring normal gait symmetry [36,38,41,42] or joint mobility [36,38]. Differences among study protocols, disease severity and treatment parameters complicate direct comparison and highlight the need for standardization. Evidence from human medicine indicates modest short-term pain and function improvements following ESWT for non-refractory knee osteoarthritis, typically within the first 4–12 weeks, with inconsistent effects occasionally persisting for up to one year [89,90,91,92,93,94,95,96,97]. While a one-year effect is considered modest in human research, an improvement of this duration would represent a meaningful clinical benefit in dogs with refractory disease. Collectively, the available canine and human data suggest that ESWT may offer short-lived symptomatic relief with limited clinical impact in dogs with advanced or treatment-refractory osteoarthritis.

Several limitations should be acknowledged. The study population was small, consistent with the exploratory character of most ESWT and physical-modality trials in dogs [36,41,42]. Joint involvement and osteoarthritis severity were heterogeneous, complicating the interpretation of symmetry-based gait variables, and many dogs had end-stage disease with a mechanically constrained range of motion, which may have limited detectable treatment effects. Furthermore, owner familiarity with chronic functional deficits may have influenced owner-reported assessments, and missing validation for clinical metrology instruments for treatment-refractory populations in dogs and humans restricts interpretation. Follow-up was limited to three months, providing only short-term information, and optimal ESWT dosing parameters remain undefined. These limitations should be considered when interpreting the findings of this study.

## 5. Conclusions

This randomized, double-blinded trial evaluated ESWT in dogs with treatment-refractory elbow or stifle OA and identified only small, inconsistent improvements, primarily in selected kinetic parameters. No treatment-associated changes were observed in joint ROM or owner-reported outcomes. ESWT was well tolerated, and repeated treatment produced slightly more consistent effects, although clinical relevance remained limited in this severely affected population. Larger studies with stratified cohorts, optimized treatment parameters, and extended follow-up are needed to define the therapeutic role of ESWT in canine OA and to determine whether earlier disease stages may exhibit a more favorable response.

## Figures and Tables

**Table 1:** Clinical Metrology Instruments: Mean values with 95% confidence intervals of Client-Specific Outcome Measures (CSOM) and Canine Brief Pain Inventory (CBPI_Pain_, CBPI_Activity_, and CBPI_Total_) for Group E and Group L at each time point. Values are expressed as absolute scores. Within-group comparisons between time points are reported as *p*-values, and statistically significant differences (*p* < 0.05) are indicated by an asterisk.

CMI	Group	Day 0	Day 14	Day 28	Day 56	Day 84	*p*-Value (Day 0–14)	*p*-Value (Day 0–28)	*p*-Value (Day 0–56)	*p*-Value (Day 0–84)
CSOM	Group E	1.50 [1.06, 1.93]	1.20 [0.77, 1.64]	1.33 [0.89, 1.76]	1.38 [0.95, 1.81]	1.34 [0.90, 1.78]	0.188	0.709	0.915	0.804
	Group L	1.46 [1.02, 1.89]	1.32 [0.89, 1.76]	1.08 [0.65, 1.52]	1.25 [0.80, 1.69]	1.22 [0.76, 1.68]	0.860	0.044 *	0.581	0.537
CBPI_Total_	Group E	3.68 [2.57, 4.79]	3.30 [2.19, 4.41]	2.86 [1.75, 3.97]	2.68 [1.57, 3.79]	2.77 [1.63, 3.91]	0.844	0.191	0.067	0.157
	Group L	3.77 [2.66, 4.88]	2.89 [1.78, 4.00]	2.50 [1.39, 3.61]	2.62 [1.48, 3.76]	3.05 [1.87, 4.23]	0.145	0.010 *	0.039 *	0.457
CBPI_Pain_	Group E	3.51 [2.66, 4.35]	3.20 [2.36, 4.05]	2.53 [1.69, 3.38]	2.27 [1.42, 3.11]	2.67 [1.80, 3.54]	0.871	0.019 *	0.001 *	0.093
	Group L	2.84 [2.00, 3.69]	2.46 [1.61, 3.30]	2.02 [1.18, 2.86]	2.08 [1.21, 2.96]	2.49 [1.58, 3.41]	0.743	0.072	0.160	0.874
CBPI_Activity_	Group E	3.61 [2.50, 4.72]	3.06 [1.96, 4.17]	2.78 [1.67, 3.88]	2.62 [1.52, 3.73]	2.58 [1.44, 3.72]	0.644	0.226	0.096	0.107
	Group L	3.97 [2.87, 5.08]	2.76 [1.65, 3.86]	2.43 [1.32, 3.53]	2.38 [1.24, 3.52]	2.87 [1.68, 4.06]	0.019 *	0.001 *	0.002 *	0.109

* Significant differences between time points within each group.

**Table 2:** Goniometric and muscle measurements: Mean values with 95% confidence intervals of joint range of motion (ROM), extension, flexion, and limb circumference for Group E and Group L at each time point. Values are expressed as percentages relative to baseline (day 0). Within-group comparisons between time points are reported as *p*-values. Statistically significant differences between time points (*p* < 0.05) are indicated by an asterisk, and statistically significant differences between groups at the same time point (*p* < 0.05) are indicated by a hash.

Measurements	Group	Day 0	Day 28	Day 56	*p*-Value (Day 0–28)	*p*-Value (Day 0–56)	*p*-Value (Day 28–56)
ROM (%)	Group E	100.0 [93.2, 108]	103.0 [96.0, 110]	104.0 [96.8, 111]	0.581	0.415	0.960
	Group L	101.0 [93.0, 108]	108.0 [101.0, 116]	107.0 [99.8, 115]	0.017 *	0.053	0.905
Extension (%)	Group E	100.0 [96.8, 104]	100.0 [96.6, 104] #	101.0 [97.1, 104]	0.981	0.970	0.907
	Group L	100.0 [96.5, 104]	105.0 [101.5, 109] #	104.0 [100.4, 108]	0.0005 *	0.010 *	0.664
Flexion (%)	Group E	100.0 [91.4, 109]	97.1 [88.5, 106]	95.8 [87.2, 104]	0.695	0.479	0.936
	Group L	100.0 [91.0, 109]	108.6 [99.6, 118]	100.6 [91.6, 110]	0.059	0.988	0.084
Circumference (%)	Group E	100.0 [97.2, 103]	100.0 [97.6, 103]	103.0 [100.5, 106]	0.906	0.012 *	0.036 *
	Group L	100.0 [96.9, 103]	101.0 [97.5, 104]	102.0 [98.7, 105]	0.867	0.306	0.600

* Significant differences between time points within each group. # Significant differences between groups at the same time point.

**Table 3:** Kinetic gait analysis: Mean values with 95% confidence intervals of Peak Vertical Pressure (PVP) and Vertical Impulse (VI) of Group E and Group L at each time point, expressed as a percentage of day 0. Mean values with 95% confidence intervals of Symmetry Index of PVP (SI_PVP_) and Ratio of PVP forelimb to hindlimb (Ratio_PVP(FL:HL)_) of both groups at each time point, expressed as absolute values. Within-group comparisons between time points are reported as *p*-values. Statistically significant differences between time points (*p* < 0.05) are indicated by an asterisk, and statistically significant differences between groups at the same time point (*p* < 0.05) are indicated by a hash.

Gait	Parameters	Group	Day 0	Day 28	Day 56	*p*-Value (Day 0–28)	*p*-Value (Day 0–56)	*p*-Value (Day 28–56)
Walk	PVP (%)	Group E	100.0 [97.0, 103]	99.7 [96.7, 103]	102.1 [99.1, 105]	0.978	0.231	0.158
		Group L	100.0 [97.0, 103]	100.4 [97.4, 103]	98.7 [95.7, 102]	0.958	0.569	0.402
	VI (%)	Group E	100.0 [96.0, 104]	99.4 [95.4, 103]	99.2 [95.2, 103]	0.924	0.861	0.989
		Group L	99.9 [95.9, 104]	102.3 [98.3, 106]	96.7 [92.7, 101]	0.239	0.073	0.0004 *
	SI_PVP_ (%)	Group E	2.35 [−3.44, 8,15]	5.56 [−0.24, 11.35]	3.12 [−2.67, 8.91]	0.023 *	0.804	0.111
		Group L	6.78 [0.99, 12.57]	3.23 [−2.56, 9.03]	5.66 [−0.13, 11.46]	0.010 *	0.629	0.113
	Ratio_PVP(FL:HL)_	Group E	1.61 [1.49, 1.72]	1.63 [1.51, 1.74]	1.60 [1.48, 1.72] #	0.609	0.989	0.519
		Group L	1.48 [1.36, 1.60]	1.47 [1.35, 1.59]	1.42 [1.30, 1.54] #	0.916	0.014 *	0.042 *
Trot	PVP (%)	Group E	100.0 [94.9, 105]	102.0 [97.3, 108]	106.0 [100.3, 111]	0.416	0.012 *	0.228
		Group L	100.0 [94.8, 105]	104.0 [99.2, 110]	107.0 [102.3, 113]	0.048 *	0.0002 *	0.211
	VI (%)	Group E	100.0 [97.0, 103]	102.0 [98.9, 105]	103.0 [100.1, 106]	0.301	0.044 *	0.598
		Group L	100.0 [97.1, 103]	100.0 [97.5, 104]	103.0 [100.1, 106]	0.958	0.057	0.110
	SI_PVP_ (%)	Group E	11.44 [3.49, 19.4]	15.22 [7.27, 23.2] #	15.67 [7.70, 23.6] #	0.001 *	0.0003 *	0.912
		Group L	5.26 [−2.37, 12.9]	3.51 [−4.10, 11.1] #	2.97 [−4.64, 10.6] #	0.210	0.069	0.850
	Ratio_PVP(FL:HL)_	Group E	1.40 [1.23, 1.58]	1.45 [1.28, 1.63]	1.36 [1.18, 1.53]	0.075	0.122	0.0001 *
		Group L	1.41 [1.24, 1.58]	1.50 [1.33, 1.67]	1.48 [1.31, 1.65]	0.0003 *	0.005 *	0.717

* Significant differences between time points within each group. # Significant differences between groups at the same time point.

## Data Availability

Additional research data, including further details on protocols, analytic methods, raw data, processed data, and study material, is available upon request to interested researchers.

## Abbreviations

The following abbreviations are used in this manuscript:

ESWT	Extracorporeal shockwave therapy
OA	Osteoarthritis
CBPI	Canine Brief Pain Inventory
CSOM	Client Specific Outcome Measures
BCS	body condition score
PVP	Peak vertical pressure
VI	Vertical impulse
ROM	Range of motion
TPLO	Tibial plateau leveling osteotomy
IEWG	International Elbow Working Group
SI	Symmetry index
FL	Forelimb
HL	Hindlimb
ICC	Intraclass-correlation coefficient
RMSE	Root mean square error
robustlmm	Robust linear mixed effects models
emmeans	Estimated marginal means
